# Clinical analysis of temporary pacemaker implantation in 6 children with fulminant myocarditis

**DOI:** 10.1186/s13019-024-02789-6

**Published:** 2024-05-22

**Authors:** Min Zhang, Xiaofang Cai, Yong Zhang

**Affiliations:** 1 The Children’s Heart Center, 100 Hongkong Road, Jiangan District, Wuhan, Hubei China; 2 Emergency Department, Wuhan Children’s Hospital, Wuhan Women and Children Medical Care Center, Tongji Medical College, Huazhong University of Science & Technology, 100 Hongkong Road, Jiangan District, Wuhan, Hubei China

**Keywords:** Fulminant myocarditis, Temporary pacemaker, Permanent pacemaker, Atrioventricular block

## Abstract

**Background:**

There is little literature on the use of temporary pacemakers in children with fulminant myocarditis. Therefore, we summarized the use of temporary cardiac pacemakers in children with fulminant myocarditis in our hospital.

**Methods:**

The clinical data of children with fulminant myocarditis treated with temporary pacemakers in Wuhan Children’s Hospital from January 2017 to May 2022 were retrospectively analyzed.

**Results:**

A total of 6 children were enrolled in the study, including 4 boys and 2 girls, with a median age of 50 months and a median weight of 15 kg. The average time from admission to pacemaker placement was 2.75 ± 0.4 h. The electrocardiogram showed that all 6 children had third-degree atrioventricular block (III°AVB). The initial pacing voltage, the sensory sensitivity of the ventricle and the pacing frequency were set to 5–10 mV, 5 V and 100–120 bpm respectively. The sinus rhythm was recovered in 5 patients within 61 h (17–134) h, and the median time of using temporary pacemaker was 132 h (63–445) h. One of the children had persistent III°AVB after the temporary pacemaker. With parental consent, the child was fitted with a permanent pacemaker on the 12th day of his illness.

**Conclusions:**

When fulminant myocarditis leads to severe bradycardia or atrioventricular block in children, temporary pacemakers have the characteristics of high safety to improve the heart function.

Fulminant myocarditis, a fatal inflammatory disease of the cardiac muscle, often results in decreased cardiac systolic function or conduction disturbances. When fulminant myocarditis affects the function of cardiac autorhythmic cell or conduction system, severe bradycardia or atrioventricular block may occur. At this point, a temporary pacemaker needs to be installed which is a kind of non-permanent electronic device and has been used more and more in children with acute and critical cardiovascular diseases [1]. There are few studies on temporary pacemakers in children with fulminant myocarditis. Therefore, we retrospectively analyzed the clinical data of children with fulminant myocarditis treated with temporary pacemaker in Wuhan Children’s Hospital from January 2017 to May 2022, and summarized the experience of the application of temporary pacemaker in fulminant myocarditis.

## Methods

This study retrospectively analyzed children diagnosed with fulminant myocarditis and treated with temporary pacemakers at Wuhan Children’s Hospital from January 2017 to May 2022. Demographic and baseline characteristics (age, sex, weight), clinical characteristics, laboratory tests (creatine kinase myocardial band, hypersensitive cardiac troponin T, N-terminal pro-B type natriuretic peptide), and echocardiography were obtained from the electronic medical record system. Temporary pacemaker data included time of pacemaker implantation after admission, pacing parameters, working hours, and clinical outcomes after implantation.

### Statistical analysis

The SPSS 22.0 software was used for data processing, in which the count data was represented by case number and percentage. The continuous variable of non-normal distribution was denoted by the median (interquartile range) and the continuous variable of the normal distribution was denoted by the mean ± standard deviation.

## Results

A total of 6 children were included in this study, including 4 boys and 2 girls, whose age distribution was between 4 and 91 months, with a median age of 50 months, and weight distribution between 10 and 21 kg, with a median weight of 15 kg.

All 6 children received methylprednisolone and immunoglobulin to modulate immunotherapy and actively performed an electrocardiogram after admission. All were diagnosed with III°AVB, and two children had concurrent ventricular tachycardia. The clinical symptoms of the 6 children were weakness in 5 cases, vomiting in 4 cases, and syncope and convulsions in one case each. Heart rate at admission ranged from 0 to 79 beats/minute, with a mean heart rate of 55 beats/minute. There were 3 cases of cardiogenic shock and 3 cases of Adams-Stokes syndrome. Echocardiography was performed on admission in all 6 children. The mean left ventricular ejection fraction (LVEF) was 53.0 ± 6.7% and the mean shortening fraction (LVFS) was 28.8 ± 8.1%. The range of CK-MB values at admission was 50 to 1636 U/L, with a median of 78 U/L. The values of hs-cTnT ranged from 0.031 to 6.62 ng/ml, with a median of 2.77 ng/ml. NT-proBNP levels ranged from 2745 to 9000 pg/ml, with a median of 9000 pg/ml. The average time from admission to temporary pacemaker placement was 2.7 ± 0.4 h. All pacemaker modes were set to VVI mode. The initial stimulation voltage, sensory sensitivity, and stimulation frequency were set at 5–10 mV, 5 V, and 100–120 beats per minute, respectively. After temporary pacemaker placement, sinus rhythm was gradually restored within 61 h (17–134) hours in 5 patients, and the median time of temporary pacemaker use was 132 h (63–445) hours. A permanent pacemaker was installed in one patient whose electrocardiogram remained III°AVB on the 12th day of illness (Table 1).

**Table 1:** Clinical data and of 6 children with fulminant myocarditis

	Patient 1	Patient 2	Patient 3	Patient 4	Patient 5	Patient 6
Age (month)	69	91	21	81	32	4
Weight (Kg)	21	21	9.5	17	13	10
Heart rate at admission(bpm)	78	27	79	56	54	0
Indication for inserting the temporary pacemaker	Cardiogenic shock + III°AVB	Cardiogenic shock + Adams-Stokes syndrome + III°AVB	III°AVB	III°AVB + ventricular tachycardia	III°AVB + Adams-Stokes syndrome	Cardiogenic shock + Adams-Stokes syndrome + III°AVB
Time of implantation upon admission(H)	3	2.5	2	3	3	3
Pacing frequency (bpm)	100	110	110	110	110	120
Restoration of sinus rhythm time (H)	86	17	NO	37	61	134
Duration of temporary pacemaker use(H)	132	88	115	165	63	445
Electrocardiogram at discharge	I°AVB	Normal	III°AVB + Pacemaker rhythm	T wave change	IRBBB	I°AVB
CK-MB (U/L)	84	50	52	139	72	1636
Hs-cTnT (ng/ml)	3.24	2.3	0.031	3.89	0.486	6.62
NT-proBNP (pg/ml)	9000	9000	2754	9000	9000	9000
EF	60	51	61	49	55	43
FS	35	25	31	24	27	20
Methylprednisolone	10 mg/kg, qd *5d	10 mg/kg, bid *5d	10 mg/kg, bid *2d	10 mg/kg, qd *5d	20 mg/kg, qd *4d	20 mg/kg, qd *3d
Immunoglobulin	2 g/kg	2 g/kg	2 g/kg	2 g/kg	2 g/kg	2 g/kg
Antiarrhythmic drugs before the temporary pacemaker	Isoproterenol	Isoproterenol	Isoproterenol	Isoproterenol	epinephrine	Isoproterenol
Life support treatment	Respirator-assisted ventilation + CRRT + a temporary pacemaker	Respirator-assisted ventilation + a temporary pacemaker	First a temporary pacemaker, then a permanent pacemaker	Respirator-assisted ventilation + CRRT + ECMO + a temporary pacemaker	a temporary pacemaker	Respirator-assisted ventilation + a temporary pacemaker
Electrocardiogram at 2 years follow-up	Normal	Normal	III°AVB + Pacemaker rhythm	Normal	Normal	Complete left bundle branch block

bpm: beat per minute, AVB: Atrioventricular Block, CK-MB: Creatine Kinase myocardial band, Hs-cTnT: hypersensitive cardiac troponin T, NT-proBNP: N-terminal pro-B type natriuretic peptide, EF: ejection fraction, FS: fraction shortening, CRRT: Continuous Renal Replacement Therapies, ECMO: Extracorporeal Membrane Oxygenation

## Discussion

Fulminant myocarditis is an acute inflammatory disease of cardiac myocytes that can lead to left ventricular dysfunction, cardiogenic shock, and refractory life-threatening arrhythmias [2–4]. Aggressive treatment of hemodynamic failure and severe arrhythmias in fulminant myocarditis is critical [5, 6]. Among other things, temporary pacemakers would play a crucial role in improving some bradyarrhythmia diseases.

Temporary pacemakers provide an effective cardiac pacing rate to stabilize the heartbeat, thereby increasing cardiac output, hemodynamics, and end-organ perfusion [2]. In this study, III°AVB was present in all six children with fulminant myocarditis who underwent temporary pacemaker insertion with parental consent. In five of the six reported cases, sinus rhythm returned to normal after effective treatment with the temporary pacemaker, which was subsequently successfully removed. Only the last child suffered from III°AVB after 115 h of treatment with a temporary pacemaker and his mental health was still poor, the child was finally implanted with a permanent pacemaker.

When fulminant myocarditis severely affects cardiac muscle cells, autorhythmic cells and conduction system, combination treatment can improve the cure rate of the disease. A life support-based comprehensive treatment regimen (LSBCTR) proposed by DW Wang includes mechanical life support, intravenous infusion of glucocorticoids and immunoglobulin, and neuraminidase inhibitors (e.g., oseltamivir), which significantly improves the cure rate of fulminant myocarditis [7–9]. ECMO can effectively support circulatory and respiratory functions until patients have sufficiently recovered. Therefore, ECMO is currently a popular choice for mechanical circulation-assisted treatment of fulminant myocarditis in children. Due to its convenience and effectiveness, ECMO is widely used in the treatment of fulminant myocarditis in children [10, 11]. The last child we report was hospitalized in the intensive care unit for two days because of vomiting, fever, and weakness and was diagnosed with III°AVB and ventricular tachycardia. Methylprednisolone and immunoglobulin were used concomitantly, and the child had a temporary cardiac pacemaker inserted within 3 h of admission. However, after medical therapy and electrical defibrillation, ventricular tachycardia still existed and the child’s hemodynamics were unstable. Therefore, the child underwent ECMO within 24 h of admission. After 12 h of combined treatment with ECMO and the temporary pacemaker, sinus rhythm was achieved and blood pressure remained stable. Finally, the ECMO was successfully removed after 163 h of supportive circulation.

Arrhythmic events such as atrioventricular block that occur in fulminant myocarditis are transient events that occur after cardiomyocytes become swollen or damaged. After active and effective treatment, the function of some damaged cardiomyocytes gradually recovered, the subsequent sinus rhythm persisted, and the atrioventricular block disappeared. Therefore, we have summarized the time to return to sinus rhythm and the total time to insertion of the temporary pacemaker in the table. On this basis, we can roughly understand the recovery time of damaged cardiomyocytes in children with fulminant myocarditis and serve as a reference for assessing the total time required for the use of temporary pacemakers.

The onset of fulminant myocarditis is rapid and severe. Therefore, early detection and active treatment are the key to improving the cure rate. Temporary pacemakers have important clinical value for patients with hemodynamic instability due to bradyarrhythmia. When temporary pacemakers are still unable to correct hemodynamic stability, combined ECMO therapy can effectively improve outcomes, thereby improving children’s prognosis.

## Data Availability

The datasets used and analyzed during the current study are available from the corresponding author on reasonable request.

## Abbreviations

III°AVB: III° atrioventricular block
CK-MB: creatine kinase myocardial band
hs-cTnT: hypersensitive cardiac troponin T
NT-pro BNP: N-terminal pro-B type natriuretic peptide
